# Supplementary material for: Work–Life Balance: Weighing the Importance of Work–Family and Work–Health Balance
Source: Int J Environ Res Public Health. 2020 Feb 1;17(3):907. doi: 10.3390/ijerph17030907 (PMC7037206; doi:10.3390/ijerph17030907)
Supplement: Supplementary file 1 [file ijerph-17-00907-s001.pdf]

Supplementary material

Harman's single factor test

jmv::efa(data = data, vars = vars(WAI 1,WAI 2,WAI 3,WAI 4,WAI 5,WAI 6,WAI 7,WAI 8,WAI 9,WAI 10,WFC 1,WFC 2,WFC 3,WFC 4,WFC 5, WFC 6, WHB 1, WHB 2, WHB 3, WHB 4, WHB 5, WHB 6, WHB 7, WHB 8, WHB 9, WHB 10, WHB 11, WHB 12, WHB 13, WHB 14, WHB 15, WHB 16, WHB 17, JS), rotation = "none", factorSummary = TRUE)

Exploratory Factor Analysis

Table S1. Factor Loadings of the EFA performed for the Harman's single factor test

	Factor						
	1	2	3	4	5	6	Uniqueness
WAI 1			0.493				0.649
WAI 2			0.350				0.783
WAI 3	0.341		0.521				0.604
WAI 4	-0.451						0.627
WAI 5	-0.477						0.656
WAI 6							0.849
WAI 7	0.306						0.781
WAI 8	0.594						0.503
WAI 9	0.585		0.324				0.502
WAI 10	0.557		0.308				0.521
WFC 1	-0.502						0.452
WFC 2	-0.635	0.325				0.304	0.349
WFC 3	-0.515						0.548
WFC 4					0.491		0.633
WFC 5	-0.360				0.493		0.451
WFC 6	-0.339				0.490		0.493
WHB 1	-0.625						0.508
WHB 2	-0.743	0.325					0.207
WHB 3	0.605						0.490
WHB 4	0.712						0.341
WHB 5	-0.591	0.317					0.453
WHB 6	-0.691		0.303				0.335
WHB 7	0.518	0.308					0.508

Table S1. Factor Loadings of the EFA performed for the Harman's single factor test

	Factor						
	1	2	3	4	5	6	Uniqueness
WHB 8	0.515			0.336			0.499
WHB 9				0.355			0.710
WHB 10	0.321			0.340			0.677
WHB 11	0.556	0.375					0.494
WHB 12	0.545	0.324		0.335			0.419
WHB 13	0.584	0.524					0.348
WHB 14	0.694	0.482					0.242
WHB 15	0.661	0.494		-0.328			0.207
WHB 16	0.660	0.487		-0.307			0.215
WHB 17	0.554	0.395					0.465
JS	0.559						0.612

Note. 'Minimum residual' extraction method was used in combination with a 'none' rotation

Factor Statistics

Table S2. Percentage of variance explained by the factors

Factor	SS Loadings	% of Variance	Cumulative %
1	9.287	27.32	27.3
2	2.857	8.40	35.7
3	1.532	4.51	40.2
4	1.266	3.72	43.9
5	1.074	3.16	47.1
6	0.855	2.52	49.6

Selection of control variables

```
require(MASS)
```

```
## Loading required package: MASS
```

```
contr<-
lm(Soddisfazione~Età+Genere+Istruzione+RelazioneAffettiva+Figli+Ruolo+Contratto+Fu
ll_PartTime,data1)
summary(contr)
```

```
##
## Call:
## lm(formula = Soddisfazione ~ Età + Genere + Istruzione + RelazioneAffettiva +
##     Figli + Ruolo + Contratto + Full_PartTime, data = data1)
##
## Residuals:
## <Labelled double>: soddisfazione lavorativa. - Sono complessivamente soddifatto
del lavoro che svolgo.
##      Min       1Q   Median       3Q      Max
## -2.74038 -0.53274  0.27206  0.51162  1.76365
##
## Labels:
##  value                            label
##      1                      1. PER NIENTE
##      2                   2. INSODDISFATTO
##      3 3 NÉ SODDISFATTO NÉ INSODDISFATTO
##      4                     4. SODDISFATTO
##      5      5. COMPLETAMENTE SODDISFATTO
##
## Coefficients:
##                         Estimate Std. Error t value Pr(>|t|)
## (Intercept)             4.346584   0.554846   7.834 8.26e-14 ***
## Età                    -0.012433   0.006135  -2.026   0.0436 *
## GenereWomen             0.063089   0.112286   0.562   0.5746
## Istruzione2             0.115427   0.238615   0.484   0.6289
## Istruzione3             0.168845   0.257967   0.655   0.5133
## Istruzione4             0.283846   0.321047   0.884   0.3773
## RelazioneAffettivaNo    0.314317   0.134687   2.334   0.0203 *
## FigliNo                -0.306835   0.138243  -2.220   0.0272 *
## Ruolo2                 -0.484628   0.367556  -1.319   0.1883
## Ruolo3                 -0.514379   0.350377  -1.468   0.1431
## Ruolo4                 -0.428815   0.405113  -1.059   0.2907
## Ruolo5                 -0.567063   0.437836  -1.295   0.1963
## Contratto2              0.024333   0.156549   0.155   0.8766
## Contratto3              0.051876   0.270385   0.192   0.8480
## Full_PartTime2          0.166996   0.155428   1.074   0.2835
## ---
## Signif. codes:  0 '***' 0.001 '**' 0.01 '*' 0.05 '.' 0.1 ' ' 1
##
## Residual standard error: 0.9238 on 300 degrees of freedom
##   (3 observations deleted due to missingness)
## Multiple R-squared:  0.05804,    Adjusted R-squared:  0.01408
## F-statistic:  1.32 on 14 and 300 DF,  p-value: 0.1938
```

```
controlselection<-stepAIC(contr,direction = "backward")
```

```
## Start:  AIC=-35.3
## Soddisfazione ~ Età + Genere + Istruzione + RelazioneAffettiva +
##     Figli + Ruolo + Contratto + Full_PartTime
##
##                      Df Sum of Sq    RSS     AIC
## - Ruolo               4    2.0157 258.04 -40.831
## - Istruzione          3    0.7839 256.81 -40.338
## - Contratto           2    0.0438 256.07 -39.247
## - Genere              1    0.2694 256.29 -36.970
## - Full_PartTime       1    0.9852 257.01 -36.092
## <none>                           256.02 -35.301
## - Età                 1    3.5045 259.53 -33.019
## - Figli               1    4.2042 260.23 -32.171
## - RelazioneAffettiva  1    4.6477 260.67 -31.634
##
## Step:  AIC=-40.83
## Soddisfazione ~ Età + Genere + Istruzione + RelazioneAffettiva +
##     Figli + Contratto + Full_PartTime
##
##                      Df Sum of Sq    RSS     AIC
## - Istruzione          3    1.1661 259.20 -45.411
## - Contratto           2    0.2136 258.25 -44.570
## - Genere              1    0.1542 258.19 -42.643
## - Full_PartTime       1    1.2056 259.24 -41.363
## <none>                           258.04 -40.831
## - Età                 1    3.3797 261.42 -38.732
## - RelazioneAffettiva  1    4.7733 262.81 -37.057
## - Figli               1    5.0196 263.06 -36.762
##
## Step:  AIC=-45.41
## Soddisfazione ~ Età + Genere + RelazioneAffettiva + Figli + Contratto +
##     Full_PartTime
##
##                      Df Sum of Sq    RSS     AIC
## - Contratto           2    0.2701 259.48 -49.083
## - Genere              1    0.2857 259.49 -47.064
## <none>                           259.20 -45.411
## - Full_PartTime       1    1.7386 260.94 -45.305
## - Età                 1    3.6330 262.84 -43.026
## - RelazioneAffettiva  1    4.4109 263.62 -42.095
## - Figli               1    4.5232 263.73 -41.961
##
## Step:  AIC=-49.08
## Soddisfazione ~ Età + Genere + RelazioneAffettiva + Figli + Full_PartTime
##
##                      Df Sum of Sq    RSS     AIC
## - Genere              1    0.2654 259.74 -50.761
## - Full_PartTime       1    1.5617 261.04 -49.193
## <none>                           259.48 -49.083
## - Figli               1    4.3588 263.83 -45.835
## - RelazioneAffettiva  1    4.4172 263.89 -45.765
```

```
## - Età                   1    4.4490 263.92 -45.727
##
## Step:  AIC=-50.76
## Soddisfazione ~ Età + RelazioneAffettiva + Figli + Full_PartTime
##
##                        Df Sum of Sq    RSS     AIC
## - Full_PartTime         1    1.3396 261.08 -51.140
## <none>                              259.74 -50.761
## - RelazioneAffettiva  1    4.3465 264.09 -47.533
## - Figli                 1    4.5883 264.33 -47.245
## - Età                   1    4.7159 264.46 -47.093
##
## Step:  AIC=-51.14
## Soddisfazione ~ Età + RelazioneAffettiva + Figli
##
##                        Df Sum of Sq    RSS     AIC
## <none>                              261.08 -51.140
## - Figli                 1    4.3785 265.46 -47.901
## - Età                   1    4.6270 265.71 -47.607
## - RelazioneAffettiva  1    4.7519 265.83 -47.459
```

```
summary(controlselection)
```

```
##
## Call:
## lm(formula = Soddisfazione ~ Età + RelazioneAffettiva + Figli,
##     data = data1)
##
## Residuals:
## <Labelled double>: soddisfazione lavorativa. - Sono complessivamente soddifatto
del lavoro che svolgo.
##      Min       1Q   Median       3Q      Max
## -2.67351 -0.54721  0.30056  0.50130  1.63095
##
## Labels:
##  value                             label
##      1                     1. PER NIENTE
##      2                  2. INSODDISFATTO
##      3 3 NÉ SODDISFATTO NÉ INSODDISFATTO
##      4                    4. SODDISFATTO
##      5      5. COMPLETAMENTE SODDISFATTO
##
## Coefficients:
##                        Estimate Std. Error t value Pr(>|t|)
## (Intercept)            4.192105   0.267281  15.684   <2e-16 ***
## Età                   -0.012965   0.005522  -2.348   0.0195 *
## RelazioneAffettivaNo  0.314381   0.132138   2.379   0.0180 *
## FigliNo               -0.304460   0.133314  -2.284   0.0231 *
## ---
## Signif. codes:  0 '***' 0.001 '**' 0.01 '*' 0.05 '.' 0.1 ' ' 1
##
## Residual standard error: 0.9162 on 311 degrees of freedom
```

```
##   (3 observations deleted due to missingness)
## Multiple R-squared:  0.03943,    Adjusted R-squared:  0.03016
## F-statistic: 4.255 on 3 and 311 DF,  p-value: 0.005768
```

Comparison of the characteristics of the sample with those of the population

Table S3. Comparison of the characteristics of the sample with those of the population of workers in north Italy

**Variable**		**Study sample (n = 318)**	**North Italy[a] (n = 13754)**
Mean age (SD)		38.14 (11.59)	44 (11.4)
Female gender		56% (177)	49%
Education level	Primary and lower secondary school	7% (22)	29%
	Upper secondary school	53% (169)	50%
	University or higher	40% (127)	21%
Type of contract	Open-ended	79% (251)	84%
	Fixed-term	17% (53)	16%
	Other	4% (14)	
Job role	Manager	3% (9)	2%
	Supervisor	13% (41)	6.5%
	White collar	72% (228)	44.2%
	Blue collar	8% (27)	46.4%
	Other	4% (13)	0.9%
Working hours	Part-time	15% (49)	21%
	Full-time	85% (269)	79%
Occupation	Chief Executives, Senior Officials and Legislators	1.3% (4)	1.2%
	Professional	11.4% (36)	12.1%
	Technicians and associate professionals	31.9% (101)	19%
	Clerical support workers	45.4% (144)	14.3%
	Service and sales workers	3.5% (11)	17.9%
	Factory worker, skilled laborer, building workers	5.3% (17)	23.9%
	Other occupations	1,2% (4)	11.8%

[a] Data from a Two-stage stratified random sample of 92071 individuals, 42047 families (Istat, 2018).

References

Istat. (2018) *Italian Labour Force Survey – October*. UniData - Bicocca Data Archive, Milan. Study Number SN202. Data file version 1.0

The jamovi project (2019). *jamovi*. (Version 1.0) [Computer Software]. Retrieved from https://www.jamovi.org.

R Core Team (2018). *R: A Language and envionment for statistical computing*. [Computer software]. Retrieved from https://cran.r-project.org/.

Revelle, W. (2019). *psych: Procedures for Psychological, Psychometric, and Personality Research*. [R package]. Retrieved from https://cran.r-project.org/package=psych.

Venables WN, Ripley BD (2002). *Modern Applied Statistics with S*, Fourth edition. Springer, New York. ISBN 0-387-95457-0, http://www.stats.ox.ac.uk/pub/MASS4.
